# CXCL12 Chemokine Expression and Secretion Regulates Colorectal Carcinoma Cell Anoikis through Bim-Mediated Intrinsic Apoptosis

**DOI:** 10.1371/journal.pone.0012895

**Published:** 2010-09-22

**Authors:** Luke J. Drury, Michael K. Wendt, Michael B. Dwinell

**Affiliations:** Department of Microbiology and Molecular Genetics, Medical College of Wisconsin, Milwaukee, Wisconsin, United States of America; National Institutes of Health, United States of America

## Abstract

**Background:**

Resistance to anoikis, apoptosis triggered by a loss of cellular adhesion to the underlying extracellular matrix, is a hallmark of metastatic cancer. Previously we have shown re-establishment of CXCL12 expression in colorectal carcinoma cells inhibits metastasis by enhancing anoikis sensitivity. The objective of these studies was to define the signaling mechanisms regulating CXCL12-mediated anoikis.

**Methodology/Principal Findings:**

Adhesion, examined by crystal violet staining, immunofluorescence microscopy, and immunoblot analysis indicated decreased focal adhesion signaling corresponding with loss of adhesion in cells constitutively simulated by CXCL12. Loss of adhesion was inhibited by pertussis toxin treatment, indicating CXCL12 regulating anoikis through G_αi_-protein coupled receptors. Non-adherent HCT116 and HT29 colorectal carcinoma cells expressing CXCL12 exhibited enhanced anoikis sensitivity by propidium iodide staining, caspase activity assays, and immunoblot compared to GFP control cells. CXCL12 producing carcinomas cultured on poly-HEMA displayed heightened Bim and loss of Mcl-1 and Bcl-2 preceding cytochrome c release, and caspase-9 activation. RNAi knockdown of Bim reversed anoikis sensitivity of CXCL12-expressing cells and fostered increased soft-agar foci formation and hepatic tumors in an orthotopic mouse model of metastasis.

**Conclusions/Significance:**

These data indicate CXCL12 provides a barrier to metastasis by increasing anoikis via activation of a Bim-mediated intrinsic apoptotic pathway. These results underscore the importance of retaining CXCL12 expression to sensitize colorectal carcinomas to anoikis and minimize tumor progression.

## Introduction

Intestinal epithelial cells migrate along the crypt-villus axis where their survival is dependent on integrin binding to the underlying extracellular matrix (ECM). At the villus apex, epithelial cells are shed into the lumen through loss of ECM contact and undergo apoptosis, a process coined anoikis [1]–[5]. Anoikis is not only essential for maintaining normal epithelial homeostasis but also provides a strong physiological barrier to cancer progression. Resistance to anoikis is a hallmark of metastatic carcinomas where cells require survival in an anchorage-independent environment such as the bloodstream to seed distant tissues [6]. The cellular and biochemical mechanisms that regulate how metastatic carcinomas lose responsiveness to anoikis remain poorly defined.

Anoikis has primarily been described as an intrinsic apoptotic pathway with cell fate being dictated through mitochondrial outer membrane permeabilization by Bcl-2 family member proteins [7]. The Bcl-2 family consists of both anti-apoptotic and pro-apoptotic proteins with the balance of these proteins regulating mitochondrial cytochrome c release. Anti-apoptotic Bcl-2 proteins such as Bcl-2, Mcl-1, and Bcl-X_L_ heterodimerize with pro-apoptotic proteins to inhibit their function [8]. Pro-apoptotic Bcl-2 family members are further characterized as multi-domain or BH3-only proteins. Multi-domain pro-apoptotic proteins, Bak and Bax, contain transmembrane domains that permeabilize the mitochondrial outer membrane to release cytochrome c resulting in activation of caspase-9-dependent apoptosis. BH3-only proteins such as Bim, Bad, Bmf, Noxa, and Puma sense apoptotic stimuli and initiate apoptosis through activation of Bak and Bax [8]. Previous studies suggest degradation of Mcl-1 modulates expression of Bim to repress anoikis [9]. Furthermore, depletion of Bim enhances anchorage-independent survival in both transformed and non-transformed cells [9], [10]. While the intracellular mediators regulating detachment-induced cell death have begun to be elucidated, the key extracellular regulators of anoikis and the Bcl-2 family of apoptotic effectors have yet to be fully defined.

There is conflicting evidence regarding the role varying secreted mediators play in regulating anoikis in non-transformed or transformed epithelia. Oncogenic proteins such as epidermal growth factor have been shown to induce anoikis resistance through regulation of Bcl-2 family members [11], while transforming growth factor beta increases anoikis [12]. Our earlier studies have shown that constitutive expression of the chemokine CXCL12 induces anoikis in colorectal carcinoma cells [13]. CXCL12 binds to its receptor CXCR4 to mediate cell-type specific physiological processes including cellular migration, survival, and apoptosis [14]–[16]. Notably, CXCL12 and CXCR4 are essential for life as mice deficient in either gene are unable to survive much past birth [17], [18]. CXCL12 was originally described as a vital chemoattractant for B cells and monocytes [19] but since has been shown to be involved in cancer progression [20], [21]. Carcinomas frequently have elevated CXCR4 expression, which is a key regulatory element in enabling tumor cell metastasis, a locomotory event characteristic of migrating immune cells [21]. Concurrently, CXCL12 protein levels are highest in common sites of metastasis including the liver, bone marrow, and lungs, suggesting that metastasis is the result of carcinomas indirectly hijacking chemokine-directed lymphocyte trafficking patterns [20]. Our work has significantly expanded the model that chemokines strictly regulate leukocyte trafficking and demonstrate that human colonic epithelial cells expressing both CXCL12 and CXCR4 play roles in epithelial restitution [22]. Subsequent work from our laboratory indicates that CXCL12 expression becomes silenced in both breast and colorectal carcinomas via DNA promoter hypermethylation and that this epigenetic repression enhances metastasis [23], [24]. In contrast to mammary carcinomas stimulated with exogenously added CXCL12 [25], we determined that re-establishment of CXCL12 expression characteristic of the normal epithelium increases anoikis sensitivity of colorectal carcinoma cells [13]. Therefore, our working model is that endogenous expression of CXCL12 by colonic carcinoma cells interrupts metastasis in part through increased anoikis sensitivity. The overarching goal of the following studies was to unravel the biochemical and cellular mechanisms through which CXCL12 induces anoikis in colonic carcinoma cells. For these studies, we took advantage of our unique colorectal carcinoma cells engineered to re-express CXCL12. Our data indicate that constitutive CXCL12 stimulation induces anoikis through an intrinsic, Bim-dependent apoptotic mechanism. These data support the model that CXCL12 production by colonic epithelial cells is a key governor limiting carcinoma progression.

## Results

### Autocrine CXCL12 reduces cellular adhesion

**Figure 1:**
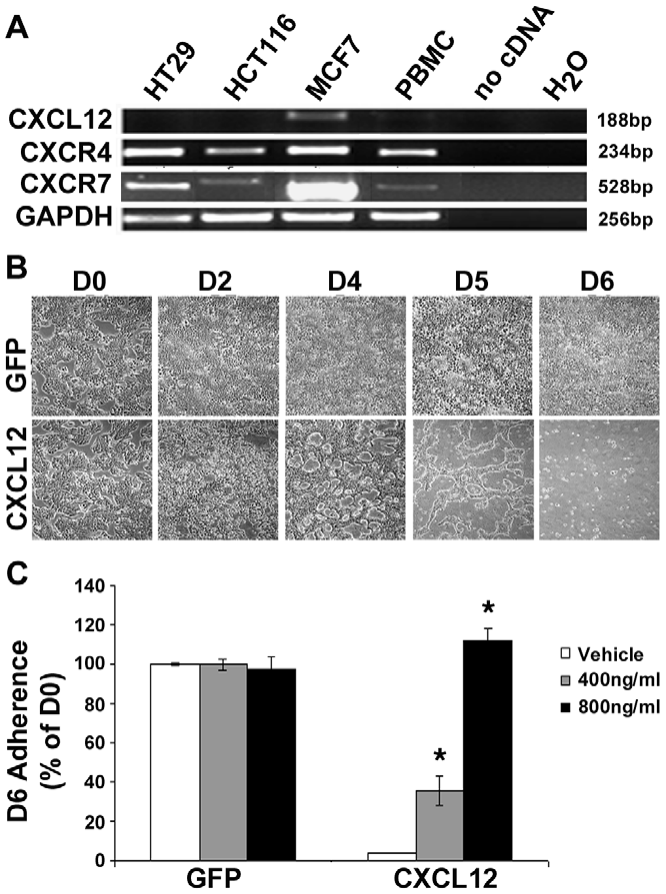
Autocrine CXCL12 decreases cell adhesion of colorectal carcinoma cells. (A) RT-PCR characterization of CXCL12, CXCR4 and CXCR7 mRNA expression in colorectal carcinoma cell lines. MCF-7 cells and peripheral blood mononuclear cells (PBMC) were analyzed as positive controls for CXCR7 or CXCR4 mRNA expression, respectively. GAPDH expression served as a loading control. (B) HT29 cells cultured in complete medium exhibit decreased adhesion after 6-days in culture (D0, D2, D4, D5, D6) compared to control (GFP) cells by phase microscopy (10X). (C) Daily administration of the G_αi_-inhibitor pertussis toxin (400 ng/ml or 800 ng/ml) restored adhesion in CXCL12-expressing cells. * indicates statistically significant difference between pertussis toxin and vehicle (DMSO)-treated CXCL12-expressing cells (*P*≤0.05).

Initial characterization of HT29 and HCT116 human colorectal carcinoma cells shows expression of both CXCR4 and CXCR7 chemokine receptors with no detectable CXCL12 expression. MCF7 breast cancer cells, previously shown to express CXCR7 and CXCL12, were used as a positive control (Figure 1A) [23], [26]. Our previous studies have shown that autocrine CXCL12 reduces cellular colorectal carcinoma cell adherence compared to wild-type colonic carcinoma cells. This decreased ability to adhere to tissue-culture-treated plastic dishes is shown for HT29 cells expressing CXCL12, which are increasingly incapable of maintaining a confluent monolayer compared to control (GFP) cells (Figure 1B). The loss of adhesion coincides with increased levels of secreted CXCL12 over that same time span, from not detectable at day 0 to a maximum after 4-days in culture (data not shown). Chemokines primarily signal through G_αi_ G-protein coupled receptors [27]. Use of pertussis toxin, a specific inhibitor of G_αi_-protein signaling, dose dependently restored cellular adhesion in CXCL12-expressing cells (Figure 1C). Reflecting our previous studies in breast cancer carcinomas, CXCR4 surface expression was unaltered between control and CXCL12-expressing cells (Supplemental Data Figure S1) [23]. These data indicate that CXCL12-mediated loss of adhesion requires activation of G_αi_-protein coupled receptors and is not a function of homologous chemokine receptor desensitization.

### Tonic CXCL12 stimulation induces breakdown of focal adhesions

**Figure 2:**
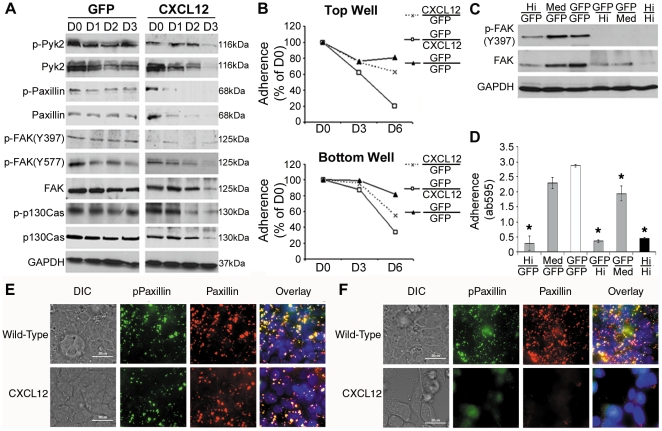
Constitutive CXCL12 stimulation induces a loss in adhesion through destabilization of focal adhesions. (A) HT29 cells expressing CXCL12 or GFP were grown to confluency as an adherent monolayer and then serum-starved three days (D0–D3). Total cell lysates were collected and immunoblot analysis conducted to examine both activation and presence of various focal adhesion proteins. GAPDH served as a loading control. CXCL12 expression resulted in a loss of focal adhesion activation, indicated by diminished phosphorylation, and loss of total protein levels. (B) CXCL12 or GFP HT29 cells were separately grown to confluency on filters or plastic and then co-cultured in serum-free medium. Adherent cells were quantified by crystal violet staining after 3 or 6 days and compared to baseline adherence at day 0. Co-cultures are identified by fractions with the numerator representing cells in the top-well insert and denominator representing cells in the bottom-well. CXCL12-expressing cells induce a decrease in cellular adhesion in control cells. (C) Lysates from cells in the top-well after six days of co-culture were collected to examine FAK activation and stability. Constitutive CXCL12 stimulation diminished active and total FAK levels whereas control (GFP/GFP) cells were unaffected. Data from panels A-C are representative of three independent experiments. (D) Transwell co-cultures with HT29 cells expressing variable levels of CXCL12 resulted in a dose dependent decrease in adhesion. Values of top-well crystal violet staining shown are mean ± SD from three independent experiments completed in triplicate. * indicates statistically significant difference in panel D from GFP/GFP co-cultured cells (*P*≤0.05). (E,F) Immunofluorescence staining and overlay (*yellow*) of active phosphorylated paxillin (*green*) with total paxillin protein (*red*) demonstrated the localization of focal adhesions in HT29 cells at day 0 (E) and day 3 (F) of adherent cultures. Size bar equals 20 µm.

With autocrine CXCL12 expression resulting in increased levels of secreted proteins and decreased adhesion we focused on focal adhesion proteins which are main mediators of cell-substrate adhesion. Focal adhesions consist of several proteins including Pyk2, paxillin, focal adhesion kinase (FAK), and p130Cas that link the cellular cytoskeleton to the extracellular matrix through transmembrane integrin proteins. Immunoblot analysis indicated that CXCL12 re-expression decreased active and total protein levels of Pyk2, paxillin, FAK, and p130Cas (Figure 2A). We determined that phosphorylation of paxillin, FAK, p130Cas, and to a lesser extent Pyk2, decreased rapidly and preceded diminishment in total cellular levels of those proteins in CXCL12-expressing cells (Figure 2A). Control (GFP) cells maintained focal adhesion protein activation and integrity. Together these data mirror our prior work investigating FAK phosphorylation of tyrosine residue 397 [13] and suggest CXCL12 expression has a broad and significant impact on proteins within the focal adhesion complex.

Given the relatively short half-life of CXCL12 [28] we sought to test the hypothesis that cellular expression of CXCL12 mediates distinct autocrine and paracrine functions relative to transient endocrine stimulation of cells. A transwell co-culture approach was used to physically separate CXCL12-expressing cells from control (GFP) HT29 cells and both cellular adherence and focal adhesion activation was ascertained. As shown in Figure 2B, CXCL12-null HT29 cells were markedly less adherent when cultured in proximity with cells continually producing the chemokine. Fluorescence microscopy confirmed that the reduced adherence of GFP control cells on inserts was not a function of chemotactic emigration toward CXCL12 secreting cells (data not shown). Consistent with data in Figure 2A, immunoblot analysis of cells cultured on inserts indicated decreased FAK phosphorylation and total protein in control HT29 cells co-cultured in proximity with CXCL12-secreting cells (Figure 2C). Moreover, HT29 cells expressing variable levels of CXCL12 (Hi and Med) demonstrated decreasing levels of total FAK protein (Figure 2C) coincident with diminished adherence (Figure 2D). The ability of CXCL12-null HT29 cells to adhere to the filter was also dose-dependently decreased when co-cultured with cells producing variable levels of the chemokine (Figure 2D). Transient 24 hr stimulation with exogenously added CXCL12 to mirror endocrine chemokine gradients was unable to recapitulate the decreased adhesion phenotype in CXCL12-null cells (data not shown). Immunofluorescence microscopy of both total and phospho-paxillin indicated intact and punctate staining consistent with focal adhesion formation in both wild-type and CXCL12-expressing HT29 cells in full growth medium (Figure 2E). Mirroring data in Figure 2A, paxillin levels were markedly decreased in CXCL12-expressing cells, compared to control, after 3-days in culture (Figure 2F). Together these data describe a role for tonic CXCL12 expression and production in regulating focal adhesion stability and cellular adherence.

### Autocrine CXCL12 expression sensitizes epithelial cells to anoikis

**Figure 3:**
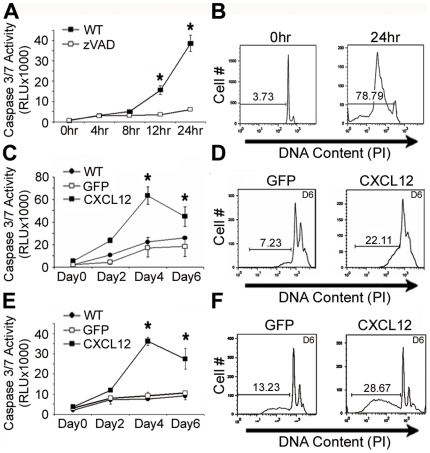
Re-establishment of CXCL12 expression enhances anchorage-independent cell death. (A, B) Non-transformed rat IEC6 intestinal epithelial cells were cultured as a suspension on poly-HEMA [10 mg/ml]-coated dishes. Caspase-3/7 activity (A) was measured using a luminescence-based assay. Execution of apoptosis was confirmed by propidium iodide staining (B) to display DNA degradation. Addition of zVAD [10 µg/ml], a pan-caspase inhibitor, blocked caspase-3/7 activity. Stable HCT116 (C, D) and HT29 (E, F) human colorectal carcinomas expressing CXCL12 or GFP (control) were plated on poly-HEMA. CXCL12 re-expression minimally altered baseline cell death at day-0 but markedly enhanced non-adherent cell death over time indicated by heightened caspase-3/7 activity and DNA degradation relative to GFP control cells. Values in panels (A–F) are mean ± SD of three independent experiments completed in triplicate. * indicates statistically significant difference between GFP and CXCL12-expressing cells (*P*≤0.05).

Our previous studies have shown that IEC6 normal intestinal epithelial cells express both CXCR4 and its ligand CXCL12 [14]. Additionally, our immunohistochemical analyses have determined that CXCL12 expression to be maximal at the crypt apex [24]. These data suggest that CXCL12 could potentially be involved in epithelial sheet turnover. To test that hypothesis, we first determined that normal, non-transformed IEC6 cells exhibit robust caspase activation and apoptosis during anchorage-independent growth (Figure 3A–B). In agreement with those data and reports of gut epithelium *in vivo* [1], HCT116 (Figure 3C–D) and HT29 (Figure 3E–F) human colorectal carcinoma cells engineered to stably express CXCL12 exhibited increased activation of executioner caspases-3/7 compared to CXCL12-deficient carcinoma cells. Propidium iodide staining confirmed DNA degradation and increased apoptosis in those cells producing CXCL12 relative to control cells (Figure 3B,D,F). Caspase-3/7 activation in CXCL12 cells was sensitive to the broad-spectrum caspase-inhibitor zVAD, consistent with apoptosis (Figure 3A,C,E). Maximal apoptotic cell death of non-adherent cultures was noted at day four in carcinoma cells relative to 24 hrs in non-transformed cells. These data suggest that CXCL12 expression sensitizes colorectal carcinomas to anchorage-independent cell death characteristic of the non-transformed epithelium.

### CXCL12 utilizes intrinsic apoptotic machinery in anoikis

**Figure 4:**
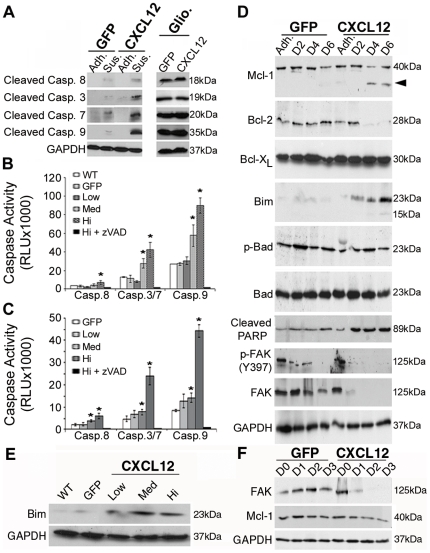
Constitutive CXCL12 expression induces anoikis through an intrinsic apoptotic mechanism. (A) CXCL12 and GFP HT29 cells were grown for 4-days as a suspension on poly-HEMA [10 mg/ml]-treated plates (Sus.) and whole cell lysates collected and compared to cells grown as an adherent monolayer (Adh.). Active cleaved caspase-8, -3, -7, and -9 indicated the predominant activation of an intrinsic apoptotic pathway in CXCL12 cells relative to control. Gliotoxin [2 µg/ml] confirmed intact apoptotic machinery in both cell lines. (B,C) Luminescence-based caspase activity assays were used to examine levels of both initiator caspases-8 and -9 and executioner caspases-3/7 after 4-days on poly-HEMA. Clonal HT29 (B) and HCT116 (C) cell lines expressing variable levels of CXCL12 (Hi, Med, Low) indicated a dose dependent effect of caspase activity which was inhibited with addition of zVAD. Values are mean ± SD of 3 independent experiments completed in triplicate. (D) GFP and CXCL12 HT29 cells were cultured on poly-HEMA-treated plates and lysates collected over time. Immunoblot analysis revealed a decrease in Mcl-1 and Bcl-2 levels and an increase in Bim in CXCL12-expressing cells. Arrowhead indicates a lower molecular weight protein consistent with an Mcl-1 degradation product. (E) HT29 cells expressing variable levels of CXCL12 exhibit increased Bim expression after a 4-day culture on poly-HEMA. (F) Immunoblot analysis of adherent HT29 cells demonstrate the temporal loss of FAK activation precedes decreased Mcl-1 levels in CXCL12-expressing cells cultured as an adherent monolayer on tissue culture plastic. Data are representative of three independent experiments. * indicates statistically significant differences in caspase activity between GFP and CXCL12-expressing cells (*P*≤0.05).

As both intrinsic and extrinsic apoptotic mechanisms have been implicated in detachment-induced cell death [2], we next investigated the apoptotic mechanism regulating anoikis sensitivity in response to tonic CXCL12 production. As a first step, we examined activity levels of the extrinsic apoptotic initiator, caspase-8, and intrinsic apoptotic initiator caspase-9 levels in carcinoma cells secreting varying amounts of CXCL12 (Hi, Med, Low) [24]. Immunoblot analysis indicated increased active, cleaved caspase-9 levels solely in non-adherent CXCL12-expressing cells compared to active caspase-8, suggesting an intrinsic apoptotic mechanism driving anoikis of those cells (Figure 4A). Gliotoxin, a known inducer of apoptosis, confirmed equal and functionally intact apoptotic machinery in GFP and CXCL12-expressing cells (Figure 4A). Consistent with those data, initiator and executioner caspase activities were measured by specific glo-assays. HT29 (Figure 4B) and HCT116 (Figure 4C) cells producing varying levels of CXCL12 demonstrated a dose-dependent and preferential increase in caspase-9 activity relative to caspase-8 after four days of adherent cell culture, consistent with the activation of an intrinsic apoptotic mechanism.

Intrinsic apoptosis is largely governed by the balance of Bcl-2 family members that dictate cell fate [8]. Congruent with our analysis of initiator caspase activity, we investigated the levels of several Bcl-2 members in non-adherent HT29 cells expressing CXCL12 to define which protein(s) were regulating apoptosis. As shown in Figure 4D, immunoblot analysis showed a marked loss of Mcl-1 and Bcl-2 expression coincident with the marked increase in Bim protein levels in anoikis sensitive CXCL12- expressing cells. Mcl-1 and Bcl-2 expression was sustained in anoikis-resistant control cells cultured in suspension on poly-HEMA. Bcl-X_L_ and Bad protein levels were unchanged between adherent and non-adherent CXCL12 and control cells. Cleaved PARP confirmed increased apoptosis in cells producing the chemokine (Figure 4D). Interestingly, decreased FAK activation and protein levels preceded the alterations in Mcl-1 and Bim levels (Figure 4D). Bim protein expression was markedly increased in three separate clones of CXCL12 expressing cells, consistent with its key role in anoikis-sensitivity and decreased metastatic potential in those cells (Figure 4E). Analysis of adherent HT29 cells confirmed that loss of FAK activation preceded decay in Mcl-1 protein levels (Figure 4F) observed in cells in suspension (Figure 4D). Consistent with its key role in anoikis, Bim was undetectable in these adherent culture time points (data not shown), further supporting the notion that loss of focal adhesions and decreased adherence precede Bim upregulation. Together, these data indicate that persistent CXCL12 stimulation induces anoikis by shifting the balance between Bim and Mcl-1/Bcl-2 levels to activate caspase-9 and intrinsic apoptosis.

### CXCL12 induces cytochrome c release via Bak upregulation

**Figure 5:**
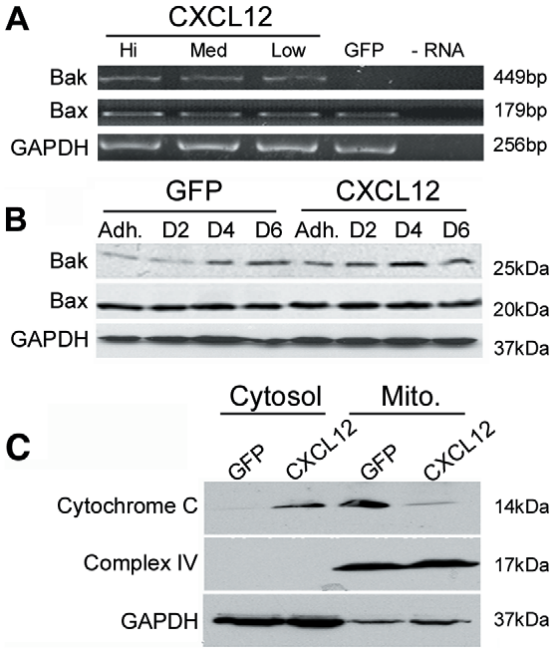
Bak expression is upregulated in non-adherent colorectal carcinoma cells expressing CXCL12. (A) HT29 stable cell lines expressing varying levels of CXCL12 were plated for 4-days on poly-HEMA [10 mg/ml]. RT-PCR analysis shows an increase in Bak but not Bax transcript levels in CXCL12-expressing cells in relation to control GFP cells. (B) Immunoblot analysis confirms increased Bak protein levels in CXCL12-expressing cells cultured in suspension for either 2, 4 or 6 days (D2, D4, D6) compared to control. (C) Mitochondrial fractionation and immunoblot analysis of GFP and CXCL12 cells after four days of anchorage-independent growth indicated a release of cytochrome c into the cytosol in CXCL12-expressing cells. Complex IV, a resident mitochondrial protein, confirmed efficient fractionation and GAPDH served as a loading control. Data are representative of 4 independent experiments.

Bcl-2 family members regulate the ability of Bak and Bax to permeabilize the mitochondrial outer membrane and initiate cytochrome c release into the cytosol [7]. We next sought to determine whether Bak or Bax expression was increased in anoikis sensitive CXCL12-expressing cells. RT-PCR (Figure 5A) and immunoblot (Figure 5B) analyses of HT29 cells revealed Bak expression was markedly increased in adherent and non-adherent carcinoma cells expressing CXCL12 relative to the transfection control GFP cells. Contiguous with Bak upregulation, cytochrome c release to the cytosol was observed solely in CXCL12-expressing cells whereas control cells retained cytochrome c in the mitochondria (Figure 5C). Immunoblot analysis of the resident mitochondrial protein Complex IV confirmed efficient mitochondrial fractionation. Together these results suggest constitutive CXCL12 production during anchorage-independent growth induces anoikis via Bak upregulation resulting in subsequent release of cytochrome c.

### CXCL12 expression and adhesion are unaffected by Bim repression

**Figure 6:**
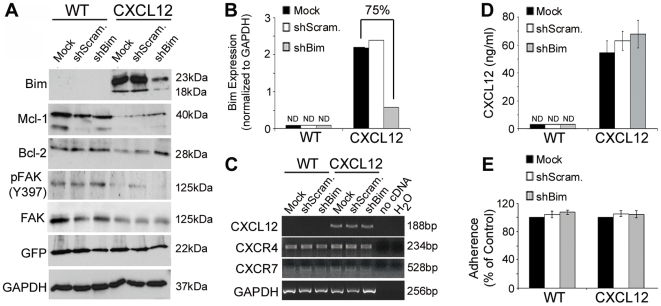
Bim depletion restores anti-apoptotic protein expression independent of changes in cellular adherence. (A) HCT116 cells were transduced by lentiviral particles encoding shRNA to create stable knockdown cell lines. Efficient knockdown of Bim levels after four days of non-adherent growth on poly-HEMA was confirmed by immunoblot analysis. Increased levels of Bcl-2 and Mcl-1 corresponded with Bim knockdown in CXCL12-expressing cells. Empty virus lacking shRNA and virus expressing a scrambled shRNA served as controls. (B) Densitometry analysis from 3 separate immunoblot analyses revealed a 75% knockdown of Bim protein levels in CXCL12 cells stably expressing Bim shRNA. (C) RT-PCR analysis confirmed CXCL12, CXCR4, and CXCR7 transcript levels were unchanged in Bim knockdown cells. (D) Enzyme-linked immunosorbent assay confirmed minimal alteration in CXCL12 secretion following lentiviral transduction. (E) HCT116 stable cell lines were grown to confluency and then serum-starved 6-days to determine if Bim depletion affected cellular adhesion. Crystal violet staining indicated adhesion was not significantly different between untransduced and RNAi-expressing cell lines. Values in D and E are mean ± SD of three independent experiments.

Having demonstrated Bim expression was upregulated in non-adherent colorectal carcinoma cells expressing CXCL12, we next examined whether RNAi-mediated repression of Bim would reverse anoikis sensitivity in those cells. Using a lentiviral system, we created wild-type and CXCL12 HCT116 cells stably expressing an shRNA sequence that specifically silences extra-long (EL), long (L) and short (S) Bim isoforms. As controls, stable cell lines expressing a scramble shRNA or an empty vector (mock) were utilized. Immunoblot (Figure 6A) and densitometry (Figure 6B) analyses of non-adherent cultures confirmed the 75% knockdown efficiency of Bim protein levels in CXCL12-expressing cells. Interestingly, Bim repression resulted in the concomitant increase in Mcl-1 and Bcl-2 levels in CXCL12-producing, but not wild-type, cells mirroring our results seen in HT29 cells (Figure 4D). FAK levels were also unaltered by Bim depletion (Figure 6A) and were consistently decreased in CXCL12-expressing cells as noted in HT29 cells. Lentiviral transduction with shRNA to Bim, scrambled transcript, or empty (mock) vectors did not modulate ligand or receptor transcript expression (Figure 6C) nor CXCL12 secretion (Figure 6D) in our colonic carcinoma cell lines. Next, we determined that Bim knockdown had little to no effect on cellular adherence in CXCL12 or wild-type cells compared to untransduced (mock) parental cells suggesting Bim is not regulating focal adhesions (Figure 6E). These data suggest that CXCL12 inversely governs Mcl-1 and Bcl-2 through the Bim regulator and that Bim knockdown does not modulate the ability of those cells to adhere suggesting focal adhesion signaling is working upstream of Bim.

### Bim knockdown reverses anoikis sensitivity in CXCL12-expressing cells

**Figure 7:**
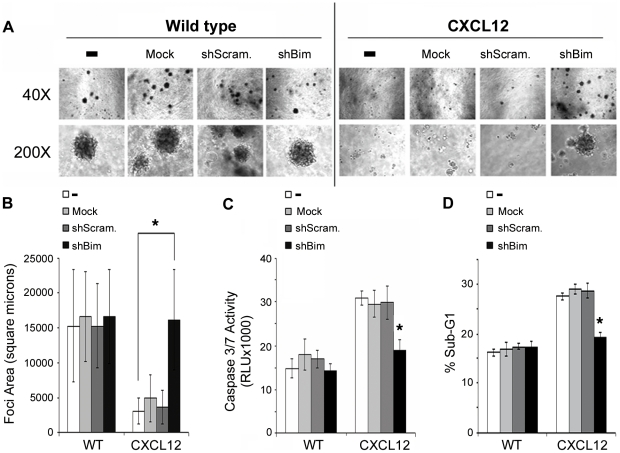
Bim knockdown rescued anchorage-independent growth in CXCL12-expressing colorectal carcinoma cells. (A, B) HCT116 stable cell lines were grown for two weeks on soft-agar to examine anchorage-independent cell growth. Foci formation was examined using bright field microscopy under 40X and 200X objectives. Bim knockdown rescued anoikis resistance in CXCL12-expressing colorectal carcinomas to equivalent levels as GFP controls. (C) Cells were cultured 4-days as a suspension on poly-HEMA treated plates and caspase-3/7 activity measured. Depletion of Bim in CXCL12 cells decreased caspase-3/7 activity to GFP control levels. (D) Sub-G1 populations indicating DNA degradation were diminished with Bim knockdown in CXCL12-expressing cells but not in control cells. Values are mean ± SD of three independent experiments completed in triplicate. * indicates statistically significant between CXCL12 untransduced and transduced cells (P≤0.05).

Bim-depleted cells were then examined to test the hypothesis that Bim regulates anoikis sensitivity in colonic carcinoma cells producing CXCL12. Previously our laboratory has shown that CXCL12-expressing colorectal carcinoma cells are impaired in both their ability to form foci on soft-agar and actively metastasize [13]. As shown in Figure 7A, Bim depletion resulted in robust soft-agar foci formation in CXCL12-expressing cells. In agreement with our prior work, soft-agar foci formation was limited in CXCL12 cells and unaffected by lentiviral transduction with empty (mock) or scrambled shRNA (Figure 7A). Carcinoma foci were of equal size in CXCL12-expressing cells depleted of Bim as chemokine-deficient wild-type HCT116 cells (Figure 7B). Consistent with soft-agar survival, knockdown of Bim in CXCL12-expressing cells significantly decreased executioner caspase-3/7 activity (Figure 7C) and DNA degradation (Figure 7D) in HCT116 cells cultured in suspension. Together these data suggest that constitutive CXCL12 expression and production inhibits tumor formation by sensitizing colorectal carcinoma cells to anoikis via upregulation of the Bim regulator.

### Bim knockdown enhances metastatic potential of CXCL12-expressing cells

**Figure 8:**
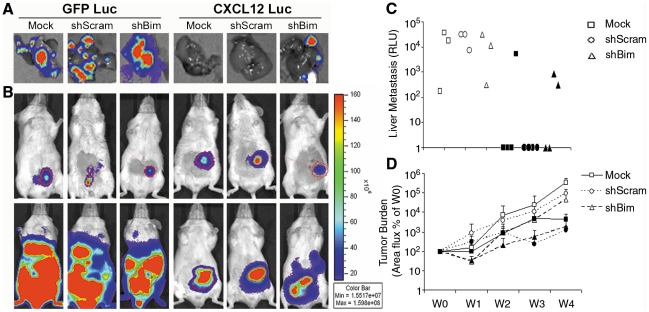
Bim knockdown partially restores metastasis of CXCL12-expressing colorectal carcinoma cells. HCT116 cells expressing either GFP and Firefly luciferase (GFP-Luc) or CXCL12 and Firefly luciferase (CXCL12-Luc) were orthotopically xenografted to the cecal wall of SCID mice. Tumor formation and hepatic metastases was monitored in real-time using biophotonic imaging. (A) Five-weeks after engraftment, GFP-Luc carcinoma cells consistently metastasized to the liver, as measured ex vivo. CXCL12-Luc cells were markedly less metastatic as shown in excised liver from mice engrafted with CXCL12-expressing cells transduced with empty lentiviral particles (mock) or particles encoding non-specific shRNA sequences (shScram). Bim depleted CXCL12 cells successfully metastasized to the liver. (B) Equivalent levels of HCT116 GFP-Luc and CXCL12-Luc cells were engrafted to the cecal wall as day 0 (top panels). Control cells formed larger tumors after 4-weeks growth (lower panels). Data in A and B are representative of 3–4 animals per group. (C) Liver metastases were enumerated using biophotonic imaging and demonstrated robust hepatic tumor levels in GFP-Luc (open symbols) cells compared to CXCL12-Luc (filled symbols). Data in C presented as radiance (RLU) normalized to background luminescence. (D) Overall tumor burden was unaffected by Bim depletion and was diminished, but not statistically decreased, over the 4-week (W0–W4) time-course in CXCL12-Luc cells (filled symbols) compared to GFP-Luc (open symbols) control cells. Data in D presented as radiance (RLU) normalized to initial (W0) values immediately after implantation.

To test that notion, our previously established HCT116 carcinoma cells expressing firefly luciferase and CXCL12 (CXCL12-Luc) or GFP (GFP-Luc) as control [13] were transduced with lentivirus to establish Bim knockdown cells. HCT-Luc cells transduced with empty vector (mock) or scrambled shRNA served as controls. Tumor cells were implanted in the cecal wall of immunodeficient SCID mice and tumor progression monitored over time using bioluminescence imaging. Consistent with our previous reports, GFP-Luc cells exhibited strong tumor growth with each of the animals developing liver metastases irrespective of Bim depletion (Figure 8A,B). CXCL12-Luc cells, which we have shown to be poorly metastatic, were unaffected by lentiviral transduction of mock or scramble shRNA viral particles with only one of eight animals developing a hepatic metastases. In parallel with our soft-agar experiments, knockdown of Bim in CXCL12-Luc cells partially restored metastatic potential of these cells, resulting in half of the mice developing hepatic tumors (Figure 8A and C). Mirroring our previous findings (Figure 8D), net tumor burden was slightly decreased, but not statistically different, over time in CXCL12-Luc mice compared to GFP-Luc controls and was unaffected by Bim depletion [13]. These data support the model that increased Bim expression in CXCL12-producing cells plays key roles in decreased metastatic potential relative to primary tumor growth of those colonic carcinoma cells.

## Discussion

Induction of anoikis provides a key regulatory mechanism for normal intestinal epithelial homeostasis and offers a potent limitation to tumor dissemination. Invasion and metastasis are responsible for 90% of cancer-associated mortalities with some cases of the tumor cells being more enumerated in secondary tissues than the primary growth site.[2] With resistance to anoikis being a hallmark of metastatic cancers, it is critical to understand mechanisms governing this process. In contrast to the current model that extracellular mediators primarily induce extrinsic apoptosis or repress anoikis sensitivity, our studies suggest that CXCL12 represents a novel secreted mediator of anoikis.

Although classically characterized as directors of cellular migration, chemokines have since have been shown to be involved in a broad range of cellular processes [15], [16], [19]. Recently, the homeostatic chemokine CXCL12 has been shown to be an essential mediator of cancer metastasis, with our laboratory demonstrating that silencing of endogenous CXCL12 expression establishes a pro-metastatic phenotype [20], [23], [24]. These studies suggest there are stark differences in cellular outcomes with endogenous and exogenous CXCL12 signaling. In particular, roles for CXCL12 in cell death have been controversial, with studies describing it either as an inducer or a repressor of apoptosis [13], [16], [25]. Kochetkova and colleagues showed that transient CXCL12 stimulation increased tumor metastasis by inhibiting anoikis through induction of Bmf and Bcl-X_L_ anti-apoptotic proteins in breast cancer cells [25]. Conversely, our lab has previously shown that re-establishment of endogenous CXCL12 expression inhibits metastasis and sensitizes colorectal carcinoma cells to anoikis [13]. Data herein delineate the molecular process by which CXCL12 re-expression regulates Bim and Mcl-1 to initiate anoikis in colorectal cancers. The dichotomy in CXCL12 functions in anoikis potentially reflects a difference between transient and constitutive chemokine expression, as tonic chemokine production elicited anoikis in distinct colonic carcinoma cell lines. Alternatively, as suggested by our prior report of increased proliferation in CXCL12-expressing human breast cancer cells [23], differential sensitivity to anoikis may point toward carcinoma-specific roles for CXCL12 in modulating carcinogenesis.

Mechanisms regulating anoikis in normal epithelial turnover and transformed colorectal carcinomas remains unclear. Herein, we show re-establishment of CXCL12 expression is a novel inducer of Bim and Bak in colorectal carcinoma cells and sensitizes these cells to anoikis. In normal intestinal epithelium expression of CXCL12 parallels Bak along the crypt-villus axis with maximal protein observed at the crypt apex [24], [29]. Recently, Duckworth and colleagues showed increased crypt hyperplasia and suppressed epithelial cell apoptosis in Bak-null mice signifying a critical role for Bak in normal intestinal epithelial turnover [30]. It is tempting to postulate that CXCL12 is a novel extracellular regulator of Bak and that this signaling axis is important in normal epithelial turnover. Coinciding with our data, decreased Bak and Bim expression are correlated with diminished disease prognosis suggesting downregulation of these proteins are critical for apoptotic resistance [31], [32]. Conversely, upregulation of Mcl-1 in colorectal carcinomas results in diminished efficacy of chemotherapy treatments such as 5-fluorouracil [33] and has been shown to mediate anoikis resistance in other cancer types [9], [10], [34]. Therefore, therapies aimed to restore expression levels of these pro-apoptotic Bcl-2 family members could improve patient prognosis. Our data suggest re-establishment of CXCL12 expression in colorectal carcinomas potentially serves as a target to sensitize these cells to chemotherapies while limiting metastatic progression by modulating levels of Bcl-2 family members.

**Figure 9:**
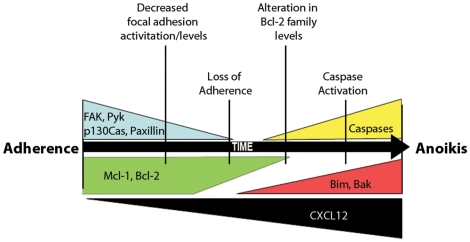
Schematic model of CXCL12-mediated anoikis in colorectal carcinoma cells. Cell culture in the presence of increasing levels of secreted CXCL12 in autologously expressing colon carcinoma cell lines reveals a temporal loss of focal adhesion proteins and increased Bim expression leading to increased anoikis and decreased metastatic potential. Initially, adherent cells expressing CXCL12 maintain expression and activation of focal adhesion proteins (FAK, Pyk, p130Cas, and paxillin) along with high levels of pro-survival Bcl-2 family members (Mcl-1 and Bcl-2). Prolonged production of CXCL12 leads to decreased focal adhesion activation and impaired cellular adhesion. Subsequently, levels of pro-survival Bcl-2 proteins decrease with the concomitant increase in pro-apoptotic Bim and Bak proteins. Changing the balance in Bcl-2 family protein expression toward increased pro-apoptotic protein levels results in caspase activation and execution of anoikis. Wedges indicate relative protein levels plotted over time.

Successful metastasis requires a degree of active focal adhesions in the context of an anoikis-resistant anti-apoptotic signaling mechanism. Our data show constitutive CXCL12 stimulation results in a loss of cellular adherence via downregulation of focal adhesions. Further, our data indicate that Bim depletion had little effect on formation or stability of focal adhesion proteins, with FAK levels decreasing equally in Bim knockdown and Bim-competent CXCL12 cells. Moreover, our analyses indicate focal adhesion protein loss of activity and degradation temporally precedes alterations in Mcl-1 and Bim expression levels, which themselves regulate activation of the caspase family members (Figure 9). These data mirror a prior report showing that epithelial anoikis reflects focal adhesion complex-dependent disruption in the balance between pro-apoptotic and anti-apoptotic Bcl-2 family members [35]. Studies with knockdown cells revealed a key role for Bim in the survivability of metastatic tumor cells expressing CXCL12 in an *in vivo* orthotopic xenograft model. The lack of an intact focal adhesion complex in those cells likely provided an additional barrier to metastasis, preventing a complete reversal in metastasis of chemokine-producing carcinoma cells. We hypothesize that maintaining focal adhesion protein expression, which is absent in CXCL12 cells regardless of Bim levels, is critical for further enabling metastasis of colorectal carcinomas expressing the chemokine.

Recently CXCL12 has been shown to bind the chemokine receptor CXCR7 [26]. In agreement with CXCR4, upregulation of CXCR7 enhances the metastatic potential of several carcinomas [26]. Interestingly, CXCR7 and CXCR4 appear to have divergent cellular functions with CXCR7 influencing cellular adhesion and CXCR4 directing migration, suggesting differential chemokine signaling mechanisms [36], [37]. However, given the unclear signaling mechanisms whereby CXCR7 mediates those effects, it is difficult to rigorously define the role for that receptor in apoptosis and tumorigenesis. Future studies will examine the role of CXCR4 and CXCR7 in CXCL12-mediated anoikis to determine if loss of adhesion and anchorage-independent cell death are differentially regulated through those receptors. It is also of interest to examine if transient and constitutive CXCL12 stimulation of either CXCR4 or CXCR7 elicits distinct cellular outcomes. Similarly, it is possible that transient ligand exposure results in homologous receptor desensitization of both CXCR4 and CXCR7, while those receptors are more resilient to more sustained receptor activation. The impact of sustained CXCR4 and CXCR7 activation on the desensitization and recycling of those receptors has yet to be explored, but may provide novel avenues with which we can specifically activate or de-activate chemokine receptors in human disease. We propose that constitutive CXCL12 stimulation more accurately represents the environment of the intestinal epithelium where cellular migration from the crypt base to the villus tip occurs along gradients of CXCL12.

Together, these data extend our understanding of CXCL12-mediated blockade of metastasis by defining mechanistically the signaling aperture utilized in anoikis. Our data indicate an intrinsic apoptotic pathway executing anoikis as evidenced by preferential caspase-9 activity and cleavage. These studies also identify previously uncharacterized interactions between CXCL12 and Bcl-2 family members in colorectal carcinomas through upregulation of Bim and downregulation of Mcl-1. Repression of Bim protein levels by RNAi rescued anoikis resistance in CXCL12-expressing cells describing Bim as a critical physiological step for colorectal carcinoma anchorage-independent survival and tumor metastasis. Together, these data suggest constitutive CXCL12 expression is a novel extracellular regulator of anoikis by inducing Bim-mediated intrinsic apoptotic pathway and inhibiting metastasis.

## Materials and Methods

### Cell lines, antibodies and reagents

HT29 (HTB-38) and HCT116 (CCL-247) human colorectal carcinoma cells and IEC6 (CRL-1592) normal rat intestinal epithelial cell lines were purchased from (ATCC Rockville, MD, USA) and maintained as previously described [14], [24]. HT29 and HCT116 cells stably expressing either GFP or varying levels of CXCL12 were previously established and described [24]. Antibodies specific for Bax, Bak, Mcl-1, Bim, phospho-Bad, total Bad, Puma, Bcl-2, Bcl-X_L_, cleaved poly-ADP ribose polymerase (PARP), cleaved caspase-3, cleaved caspase-9, cleaved caspase-8, and cleaved caspase-7 were from (Cell Signaling, Danvers, MA, USA). Polyclonal donkey anti-rabbit horseradish peroxidase (HRP)-conjugated secondary antibody (Amersham, Piscataway, New Jersey, USA) and SuperSignal West Pico Chemiluminescent Substrate (Pierce, Rockford, IL, USA) were used for visualization. Antibodies for monitoring focal adhesion proteins were: total FAK, phospho-FAK (Y397), total paxillin, total p130Cas (Cell Signaling), and phospho-paxillin (Y118), phospho-p130Cas (Y410), total-Pyk2, phospho-Pyk2 (Y402) and phospho-FAK (Y577/Y578) from (BD Biosciences, San Jose, CA, USA). Pertussis toxin was used for inhibition of G_αi_ dependent G-protein coupled receptor signaling (EMD Calbiochem, San Diego, CA, USA).

### Reverse Transcriptase Polymerase Chain Reaction

HCT116 cells (2×10^6^) were plated on poly-hydroxyethylmethacrylate (poly-HEMA) [10 mg/ml] (Sigma, St. Louis, MO, USA) in serum-free medium for four days. Cells were then collected, washed in PBS, and RNA was isolated using RNAeasy Kit (Qiagen, Valencia, CA, USA). RNA (2 µg) was converted into cDNA using random priming and Superscript-II reverse transcriptase in a 40 µl volume. Bak and Bax mRNA expression was amplified using 35 cycles of 94°C for 30 sec, 60°C for 30 sec, and 72°C for 30 sec. CXCL12, CXCR4 and CXCR7 expression were amplified using 33 cycles of 94°C for 30 sec, 60°C for 30 sec, and 72°C for 30 sec. Analysis of GAPDH expression was used to assess equality in cDNA levels between samples.

### Receptor surface-expression

Confluent HT29 cells (∼1×10^6^ cells) were detached with 20 mM EDTA in PBS, fixed 10 min at 4°C in 2% (w/v) paraformaldehyde in PBS, and subsequently incubated 90 min at 4°C with 10 µg/ml of murine monoclonal antibodies to CXCR4 (clone 12G5, R&D Systems, Minneapolis, MN, USA) or a mouse isotype matched control antibody as a control. Rabbit polyclonal antibody was used to detect CXCR7 (Abcam, Cambridge, MA, USA). Receptor staining was visualized by incubation with R-phycoerythrin labeled goat anti-mouse IgG (Southern Biotechnology Associates Inc., Birmingham, AL, USA) or AlexaFluor-488 labeled donkey anti-rabbit antibodies. Antibodies were optimally diluted in PBS, 1.0% (w/v) BSA and 1.0% (v/v) goat or donkey serum. Cells were analyzed by flow cytometry (Millipore, Billerica, MA, USA) and histograms made using FlowJo7.6 software (BD Laboratories, Sunnyvale, CA, USA).

### Immunoblot analysis

HT29 or HCT116 cells were plated to confluence in 6-well dishes and then serum-starved for varying times. Cells were solubilized and total cell lysate prepared as previously described [14]. Proteins were size separated using reducing SDS-PAGE, electrotransferred to PVDF membranes and visualized with appropriate HRP-conjugated secondary antibodies and SuperSignal West Pico Chemiluminescence substrate (Pierce).

### Propidium iodide staining

HT29 or HCT116 cells (4×10^6^) were cultured as a suspension by plating to poly-HEMA-coated 10 cm dishes in serum-free medium. After four days, cells were collected and washed in PBS. Cells were ethanol fixed and stained with 50 µg/ml propidium iodide (EMD Calbiochem) and 10 µg/ml RNAse A (Promega). Cell death was quantified by flow cytometric analysis of sub-G1 populations indicative of DNA fragmentation.

### Caspase activation assay

Cells were plated (4×10^6^) in serum-free medium on poly-HEMA-coated plates. Cells were collected at various time points, mixed at a 1∶1 volumetric ratio with glo reagent (Promega, Madison, WI, USA) and incubated at room temperature for 30 min in the dark. Caspase-3/7, -8, and -9 activation was measured using specific caspase glo reagents and quantified as luminescence units using a spectrophotometer (Victor Wallac, Turku, Finland).

### Mitochondrial fractionation and cytochrome c release

Cells were grown until confluent on 10 cm dishes and then serum-starved for four days. Total cells were collected, centrifuged, and washed twice in PBS. Using the MitoIso2 kit (Sigma), cells were resuspended in 1 ml of 1x isotonic extraction buffer with protease inhibitor cocktail (1∶100) and incubated on ice for 15 min. Cells were placed in a Dounce homogenizer and lysed. Homogenate was collected and centrifuged at 600×*g* for 10 min at 4°C. Supernatant was transferred to a fresh tube and centrifuged at 11,000×*g* for 10 min at 4°C. The cytosolic and mitochondrial fractions were separately solubilized in 200 µl lysis solution supplied by the manufacturer (Sigma). Cytochrome c release into the cytosolic fraction was analyzed by immunoblot and complex IV confirmed efficient mitochondrial protein isolation.

### shRNA lentivirus construction and transduction

Bim shRNA was generated using the Lentilox3.7 system (Addgene, Cambridge, MA, USA). The sequences for Bim shRNA oligos were as follows: 5′-tGGTTATCTTACG ACTGTTAttcaagagaTAAC AGTCGTAAGATAACCtttttggaaac-3′ and 5′-tcgagtt tccaaaaaGGTTATCTTACGACTGT TAtctcttgaaTAACAGTCGTAAGATAACCa-3′. Oligonucleotides were annealed together and cloned into HpaI/XhoI digested pLL3.7 GFP or pLL3.7 puromycin expression vectors. Plasmid orientation was verified by PCR and DNA sequencing.

LL3.7 empty, scramble, or Bim shRNA vectors were transfected along with accessory plasmids pVSVG, pREV, and pRRE into HEK293T cells using Mirus-293 transfection reagent. Medium was replaced after 24 hrs and conditioned medium containing viral particles was collected 48 hrs later. Viral particle supernatant was filtered through a 0.45 µm filter. HCT116 cells were transduced with lentiviral particles in the presence of polybrene [10 µg/ml] (Sigma) for 24 hrs. Medium was replaced and eGFP expression monitored by flow cytometry 48 hrs later or cells expressing the insert were selected with 5 µg/ml puromycin. Knockdown of Bim was confirmed by immunoblot analysis.

### Soft-agar foci formation assay

HCT116 GFP and CXCL12 stable cell lines (1×10^5^) expressing Bim or scramble shRNAs were plated to 12-well dishes on a 1.0 ml layer of 0.7% (w/v) bacto-agar (BD Bioscience) reconstituted in serum-containing medium. Cells were then covered with 1.0 ml of 0.35% (w/v) warm agar in serum-containing medium. Cells were cultured for two weeks and cellular foci were photographed and foci number and area determined by an investigator blinded to the status of the samples using Metamorph software. Ten representative images from each group were used to quantify foci area from three separate experiments.

### Adherence assay

HCT116 GFP and CXCL12 Bim shRNA or scramble control cells were grown to confluency in a 12-well dish and then serum starved four days. Cells were stained with crystal violet and solubilized in 2% (v/v) SDS. Absorbance (595 nm) was quantified using spectrophotometer (Victor Wallac) to determine levels of cell adherence after serum deprivation.

### Immunofluorescence Microscopy

Wild-type or CXCL12-expressing HT29 cells were grown to confluency on coverslips in 6-well dishes. Cells were washed and fixed with 2% (w/v) paraformaldehyde and permeabilized with 0.03% (v/v) Triton X-100. Coverslips were blocked with 1% (w/v) BSA at room temperature for 1 hr and then incubated room temperature for 2 hrs in primary antibody or isotype control immunoglobulins. Cells were washed and incubated 1 hr at room temperature with the appropriate secondary antibody, and co-stained with 4′,6-diamindino-2-phenylindole (Molecular Probes, Invitrogen) to visualize nuclei. Images were obtained using a fluorescence microscope at 600X magnification.

### Mouse bioluminescence imaging

In accordance with protocols approved by the Medical College of Wisconsin Institutional Animal Care and Use Committee, metastatic HCT-Luc cells (2×10^6^) were suspended in 50 µl sterile PBS were implanted to the subserosal layer of the cecum in eight-week-old male immunodeficient SCID (cr-Prdkc^scid^, Charles Rivers, Wilmington, MA) mice following a midline laparotomy. Tumor formation and metastasis was compared to HCT-Luc-CXCL12 (2×10^6^) cells. Mice were imaged immediately after implantation and then weekly by bioluminescence imaging (Lumina IVIS-100 *In Vivo* Imaging System, Xenogen Corp, Alameda, CA, USA). To visualize luciferase-expressing carcinoma cells, mice received an intraperitoneal injection of D-luciferin and anesthetized with isoflourane using the XGI-8 gas anesthesia system (Xenogen). Mice were imaged for 30 seconds with medium binning and an f-stop of 1 and Regions of Interest defined as radiance (photons/second/cm2/steradian) and presented as radiant luminescence units according to the manufacturer's calibration (Xenogen).

### Statistical Analyses

Data were analyzed using Student's unpaired *t*-test using SigmaPlot (Systat Software, Inc., San Jose, CA, USA). A *P*-value ≤0.05 was considered statistically significant.

## Supporting Information

Figure S1 CXCR4 and CXCR7 surface receptor localization is indistinguishable between CXCL12-expressing and chemokine null colon carcinoma cells. Surface localization was determined using flow cytometry. Upper panels. CXCR4 (A) and CXCR7 (B) cell surface levels in wild-type (WT) HT29 cells. Mean fluorescence intensity of CXCR4 (58.0±2.3) and CXCR7 (4.0±0.4). Lower panels. CXCR4 (C) and CXCR7 (D) membrane levels in CXCL12-expressing HT29 cells. Mean fluorescence intensity of CXCR4 (57.9±1.8) and CXCR7 (4.0±0.3). Values are the mean±SEM n = 3. Shaded areas represent unstained samples. Histogram data are representative of three independent experiments.
(4.15 MB TIF)
