# Supplementary figures and images for: CXCL12 Chemokine Expression and Secretion Regulates Colorectal Carcinoma Cell Anoikis through Bim-Mediated Intrinsic Apoptosis
Source: PLoS One. 2010 Sep 22;5(9):e12895. doi: 10.1371/journal.pone.0012895 (PMC2943927; doi:10.1371/journal.pone.0012895)

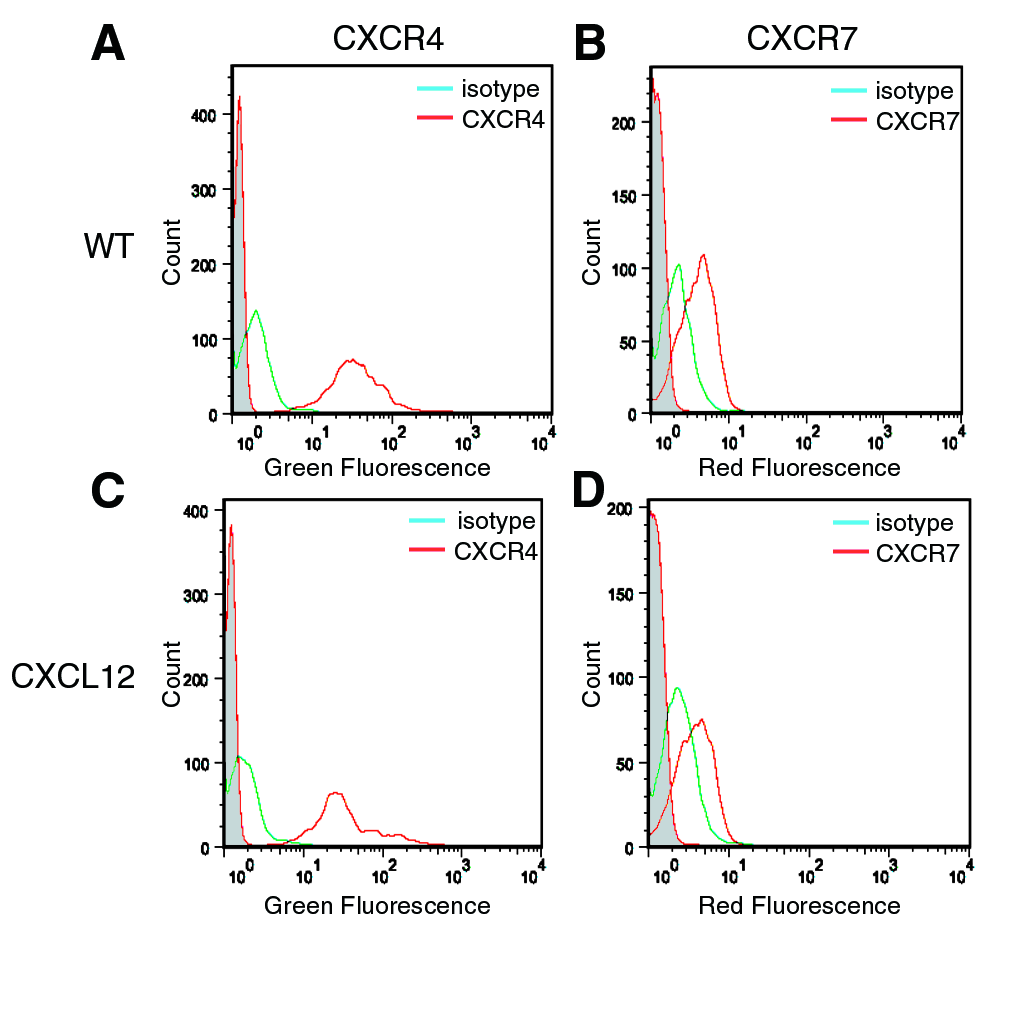

Supplement: Figure S1 — CXCR4 and CXCR7 surface receptor localization is indistinguishable between CXCL12-expressing and chemokine null colon carcinoma cells. Surface localization was determined using flow cytometry. Upper panels. CXCR4 (A) and CXCR7 (B) cell surface levels in wild-type (WT) HT29 cells. Mean fluorescence intensity of CXCR4 (58.0±2.3) and CXCR7 (4.0±0.4). Lower panels. CXCR4 (C) and CXCR7 (D) membrane levels in CXCL12-expressing HT29 cells. Mean fluorescence intensity of CXCR4 (57.9±1.8) and CXCR7 (4.0±0.3). Values are the mean±SEM n = 3. Shaded areas represent unstained samples. Histogram data are representative of three independent experiments. (4.15 MB TIF) [file pone.0012895.s001.tif]
